# LsHSP70 is induced by high temperature to interact with calmodulin, leading to higher bolting resistance in lettuce

**DOI:** 10.1038/s41598-020-72443-3

**Published:** 2020-09-16

**Authors:** Ran Liu, Zhenqi Su, Huiyan Zhou, Qian Huang, Shuangxi Fan, Chaojie Liu, Yingyan Han

**Affiliations:** 1 Beijing Key Laboratory of New Technology in Agricultural Application, National Demonstration Center for Experimental Plant Production Education, Beijing University of Agriculture, Beijing, 102206 China; 2 Laboratory of Cell & Molecular Biology, Institute of Vegetable Science, Zhejiang University, Hangzhou, 310058 China

**Keywords:** Plant development, Plant hormones, Plant stress responses

## Abstract

High temperatures have significant impacts on heat-tolerant bolting in lettuce. In this study, it was found that high temperatures could facilitate the accumulation of GA in lettuce to induce bolting, with higher expression levels of two heat shock protein genes *LsHsp70-3701* and *LsHsp70-2711.* By applying VIGS technology, these two *Hsp70* genes were incompletely silenced and plant morphological changes under heat treatment of silenced plants were observed. The results showed that lower expression levels of these two genes could enhance bolting stem length of lettuce under high temperatures, which means these two proteins may play a significant role in heat-induced bolting tolerance. By using the yeast two-hybrid technique, it was found that a calmodulin protein could interact with LsHsp70 proteins in a high-temperature stress cDNA library, which was constructed for lettuce. Also, the Hsp70-calmodulin combination can be obtained at high temperatures. According to these results, it can be speculated that the interaction between Hsp70 and calmodulin could be induced under high temperatures and higher GA contents can be obtained at the same time. This study analyses the regulation of heat tolerance in lettuce and lays a foundation for additional studies of heat resistance in lettuce.

## Introduction

Lettuce is an annual or biennial herbaceous plant in the Asteraceae family. Its beautifully shaped leaves are rich in nutrients and low in calories. Lettuce grows well in cold climates: a suitable temperature for its growth is approximately 15–20 °C, while heat stress seriously affects its growth and development. When the temperature exceeds 30 °C, lettuce tends to bolt. As the leaves of lettuce are generally the primary edible part, bolting reduces the food quality of this crop^1–3^. According to Fukuda^4^, high temperatures promote lettuce bolting. Thus, bolting resistance is a particularly desirable property in lettuce, especially in areas with hot summers or in tropical regions^5^.

High temperature stress refers to a temperature environment that causes irreversible damage to a plant when the ambient temperature exceeds the appropriate temperature for normal plant growth and development for a certain period^6^. A special group of proteins are synthesized under heat stress, and the synthesis of normal proteins is inhibited. The specific proteins synthesized by high-temperature triggered gene transcription are called heat shock proteins (HSPs)^7^. According to their different molecular weights, heat shock proteins are divided into 6 categories: Hsp100s, Hsp90s, Hsp70s, Hsp60s, small molecule Hsps and ubiquitin^8^. Under heat stresses, HSPs bind to heat-denatured proteins, participating in protein folding and preventing protein denaturation^9,10^. HSPs are related to plant heat tolerance and have been characterized through studies in soybean^11^, tobacco^12^ and potato^13^. The cytoprotective role of Hsp70 is related to the activation of protein kinases, protease activity, binding to cellular endogenous hormone receptors, and ATP hydrolysis and repair^14^. Hsp70 also plays an important role in the plant hyperthermia response. *Arabidopsis* plants lacking *AtHsp70-15* were found to die in large numbers upon heat shock^15^. According to previous studies, the thermoresistance responses of plants may be synergized in a variety of ways^16–18^.

Previous research shows that GA_3_ can significantly promote lettuce bolting, distinctly increasing the peduncle height and bolting rate^19^. GA (gibberellin) plays an important role in the regulation of lettuce stem elongation and in that of other similar plant species. Study showed that this phytohormone was regulated by environmental signals^4^. During the bolting process in radishes, there was a significant peak in the GA content at the budding point. This GA content was closely related to the bolting of buds and played a leading role in bolting^20^. After the vernalisation of cabbage, GA treatment can cause plants to bolt and flower earlier. Also, GA can accelerate the completion of bolting and flowering in vernalized cabbage plants^21^. Ca^2+^ and cGMP are positively regulated during GA signal transduction^22,23^. In rice aleurone cells, Ca^2+^ binded to calmodulin to form a Ca^2+^/calmodulin complex and regulated intracellular Ca^2+^. During GA signal transduction, the Ca^2+^/calmodulin complex can regulate Ca^2+^ ATPase, depending on the expression of GA-responsive genes^23^. Several studies have shown that the exogenous application of Ca^2+^ promoted heat tolerance in plants^18^.

In a previous study in our lab, two Hsp70 proteins were obtained by two-dimensional electrophoresis^24^. The full-length sequences of these two genes were obtained by RACE (rapid amplification of cDNA ends), and they were named *LsHsp70-3701* and *LsHsp70-2711*. Through experimental analysis, we concluded that high temperatures can promote the interaction of LsHsp70 proteins and calcium-calmodulin. We speculate that this interaction induces the accumulation of GA in the zeaxanthin pathway and thereby leads to bolting.

## Materials and methods

### Plant materials and growth conditions

The ‘*S39*’ variety was screened for typical bolting characteristics in our lab. This variety was selected in this study as it is prone to bolting and is very sensitive to high temperature. When the five-leaf stage was reached, lettuce materials were grown under the culture conditions at 25 °C, light at 20,000 lx, and a light/dark photoperiod of 16 h/8 h, with watering once every 2 days. After 1-week’s adaptation, the plants were exposed to high temperature (37 °C) (25 °C as control). After these heat treatment, after treatment, the seedlings were monitored for 0–9 days as their sampled leaves and stems were frozen in liquid nitrogen and stored at − 80 °C for RNA extraction and the determination of their endogenous hormone contents.

### Total RNA extraction and first-strand cDNA synthesis

The samples were added to liquid nitrogen and ground into powder. The total RNA was extracted by Trizol method. The extracted total RNA was used as a template for the first-strand cDNA synthesis using a kit (TransGen) with oligo (dT) as a primer. The cDNA was synthesized as a template for gene amplification and quantitative real-time PCR (qRT-PCR).

### Cloning of *LsHsp70-2711* and *LsHsp70-3701* in lettuce

The total RNA was extracted from lettuce that had been subjected to high temperature, and the cDNA sequences were reverse-transcribed. The primers were designed according to the coding sequences (CDS) of *Hsp70-3701* (COGE: Lsat_1_v5_gn_9_78021.1) and *Hsp70-2711* (COGE: Lsat_1_v5_gn_9_22920.1) (Supplementary Table S1). The CDS of *Hsp70-3701* and *Hsp70-2711* were cloned, transformed, and verified by sequencing.

### qRT-PCR expression analysis

Using the procedures for the Real Master Mix (SYBR Green) PCR kit (Clontech, Mountain View, CA, USA), a BIORAD CFX Connect Real-Time PCR System (BIORAD, USA) was used for the quantitative real-time PCR (qRT-PCR) experiments. The standard cDNA and the sample under analysis were replicated for three times. The internal reference and primer sequences were designed by Premier software as gene-specific primers (Supplementary Table S1). The expression levels in the lettuce leaves at 25 °C were set to 1, and the relative expression was calculated and plotted by using the 2^−ΔΔCt^ method.

### Construction and infection of *LsHsp70-2711* and *LsHsp70-3701* VIGS vectors

The 495 bp and 497 bp open reading frames of *LsHsp70-3701* and *LsHsp70-2711* were used to design the primers (Supplementary Table S1). Then the fragments were cloned from the ‘S39’ cDNA. The cloned fragments and pTRV2 empty vector were digested with XbaI endonuclease and KpnI endonuclease. After purification, the fragments were ligated and transformed to obtain a recombinant plasmid.

The identified recombinant plasmid was transformed into the *Agrobacterium* strain GV3101, and an infection solution was prepared. The optimal concentrations of antibiotics to add to the bacterial solution were as follows: 50 mg/l kanamycin, 50 mg/l rifampicin, 10 mM MES, and 20 mM acetosyringone. The infection buffer consisted of 10 mM MgCl_2_, 10 mM MES, and 20 mM acetosyringone. Up to four leaves of the S39’ variety were subjected to infection; 50 ml sterile syringes with the needles removed were used to push the infection solution slowly into the leaves.

After 3 weeks’ infection, the plants were held at 37 °C for 1 h and then cultured at 25 °C for 1 week. The cDNA of the young leaves was used to detect the effects by PCR and RT-PCR using gene-specific primers (Supplementary Table S1). To observe the morphological changes in the gene-silenced plants, the stem lengths of the plants which were infected for 3 weeks were measured. The plant stem length was measured 1 week after 1 h-heat-stress treatment for 3 weeks.

### Bait plasmid construction and identification

For gene cloning and construction of cloning vector, based on the existing sequences of *LsHsp70-3701* and *LsHsp70-2711* retained in our laboratory, the two genes were cloned by PCR using the listed primers (Supplementary Table S1). The restriction enzymes NdeI and SfiI was used for *LsHsp70-3701*, while the restriction endonuclease enzymes SmaI and NotI for *LsHsp70-2711*. The purified PCR products were ligated, transformed, and verified by sequencing. pTOPO-Blunt-3701 and PGBKT7 were digested with the restriction endonucleases NdeI and SfiI, respectively. The products were purified, ligated, and transformed into DH5α competent cells. Positive clones were selected and sequenced. After plasmid extraction and verification, the stocks were preserved at − 20 °C.

### Yeast two-hybrid system

The construction of yeast libraries and yeast two-hybrid screening was performed according to the instructions for the nuclear system yeast library (Invitrogen). The library was constructed with mRNA from lettuce which was subjected to high temperature. The bait plasmid was transformed into the Y_2_H GOLD strain, and the appropriate concentration of AbA (aureobasidin A) was determined by using SD-Trp/AbA plates at 75 ng/μl, 87.5 ng/μl, 100 ng/μl, 112.5 ng/μl and 125 ng/μl.

The bait plasmid, the negative control and the positive control were transformed separately into the Y_2_H GOLD strains and examined on SD-T/-L/-H plates to determine whether they were self-activated or not. After verification, the results were entered into the official COGE website for BLAST comparison, and the number of genes interacting with *LsHsp70-2711* and *LsHsp70-3701* were obtained.

### Bimolecular fluorescence complementation (BiFC)

The calmodulin gene were selected for cloning and a pSPYNE vector containing the interacting protein were obtained through seamless cloning. *LsHsp70-3701* was ligated to the pSPYCE vector and transformed (the primer designs are listed in Supplementary Table S1). The vector containing the gene of interest was transformed into competent cells of *Agrobacterium tumefaciens* EHA105 and cultured for 48 h. A single colony was picked and cultured for 8 h and sequenced. The sequencing was correct, and the strain was continued under culture for 14 h. The OD600 value was 0.4–0.5. A mixture containing pSPYCE, pSPYNE and 19p (ratio: 2:2:1) was applied, before which it was centrifuged and resuspended. When the OD_600_ value reached 0.5–0.6, the mixture could stand for 3 h. 100 μl of the bacterial liquid was injected into the tobacco leaves using a sterile syringe and observed after 48 h of culture.

### Determination of the GA content

The GA content was determined by enzyme-linked immunosorbent assay (ELISA). Kits were provided by the Agronomy and Biotechnology College of China Agricultural University. For sample processing, 0.2000 g leaves were ground in 80% methanol solution (containing 1 mmol/l BHT) and homogenized. Then it was extracted for 8 h at 4 °C and centrifuged at 4,000 r/min and 4 °C for 10 min. After the supernatant was collected, the precipitate was extracted twice more with 80% methanol. The supernatants were combined and purified on a Sep-Pack C18 column. The resulting solution was dried under nitrogen and diluted to 2 ml with PBSTG for ELISA. The ELISA results were read on a Bio-Rad Model 550 (USA) at a wavelength of 490 nm.

### Paraffin sections

Flower buds were fixed with 2.5% glutaraldehyde fixative and then dehydrated with ethanol, followed by 1/2 ethanol + 1/2 xylene → pure xylene → pure xylene transparent treatment, followed by wax. Paraffin wax and wax-permeated flower buds were poured together into small containers for embedding and then sliced and placed on slides. After drying, the slides were dewaxed and stained. Finally, the slides were sealed and photographed under a light microscope (D72, Olympus, Tokyo, Japan).

## Results

### High temperature could induce the bolting of lettuce, promote GA accumulation and increase the expression levels of *LsHsp70-3701 and LsHsp70-2711*

**Figure 1:**
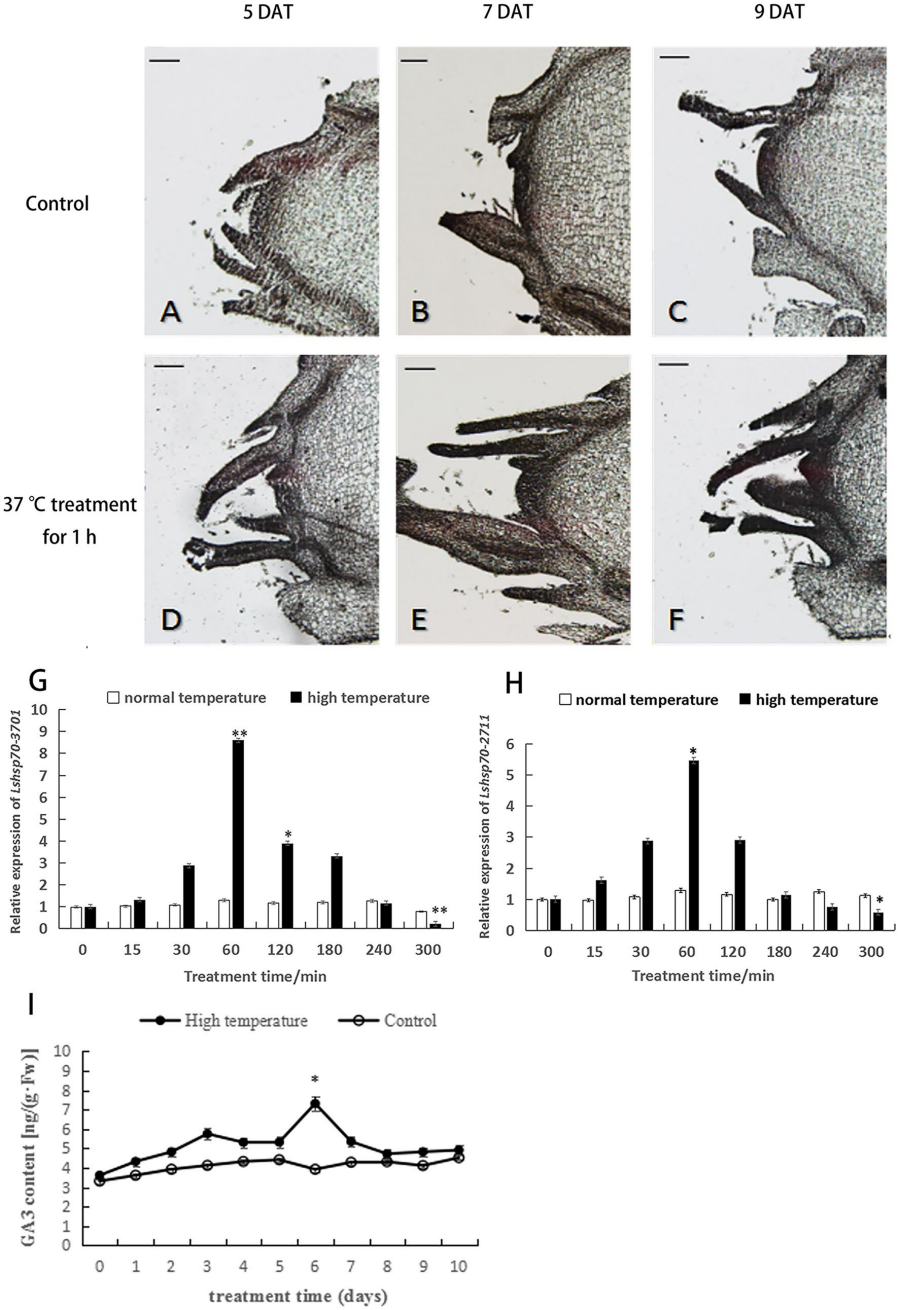
Inflorescence morphologic observation, relative expression of *LsLSP70* and GA content under high temperature treatment. (**A**–**F**) Paraffin sections of flower buds during high temperature treatment. Bars = 100 μm. *DAT* Days after treatment. (**G**,**H**) Relative expression of the *LsHsp70-3701 and LsHsp70-2711* genes at different treatment times. Significant differences were determined by Student’s t-test (∗represents P < 0.05, and ∗  ∗indicates P < 0.01). (**I**) Changes in GA_3_ hormone levels in lettuce leaves following treatment with a high temperature. Significant differences were determined by Student’s t-test (∗represents P < 0.05).

As high temperatures may cause different defects during the growth of lettuce, we chose different heat-treatment time to examine the changes in expression levels of *LsHsp70-3701* and *LsHsp70-2711*. As can be seen from the results (Fig. 1G,H), the expression levels of these two genes increased when lettuce were under heat-stress for 15, 30, 60 and 120 min; and then a decreasing pattern, both in control and high-temperature treatment. As both genes showed the highest enhanced expression levels under 60-min heat treatment, this time and condition was selected for later experiments.

On the 5th, 7th and 9th days at control temperature, the shoot apical meristem began to elongate with its shape domed, and the leaf primordium remained around the meristem for differentiation; while on the 7th day after the high-temperature treatment, the shoot apical meristem of lettuce was elongated and the top became gradually hemispherical, which indicated that the plant had started to enter the bolting stage. On the 9th day of the high temperature treatment, the growing point on the top of flower buds became larger, and the shoot apical meristem became hypertrophied. This showed that lettuce tended to bolt earlier under heat-stress than controls (Fig. 1A–F).

On the other hand, the contents of GA_3_ in the leaves of the treatment group reached a peak on the 6th day (Fig. 1I), while this content remained stable in the control group.

Together, it can be concluded that high temperatures for appropriate durations can induce GA contents and the expression levels of *LsHSp70-3701* and *LsHSP70-2711*, which indicated that GA and these two gene may play synergic or antagonistic roles in regulating the heat-induced bolting of lettuce.

### The reduction in expressions of *LsHsp70-2711* and *LsHsp70-3701* in lettuce under high temperature accelerates the elongation of lettuce stems

**Figure 2:**
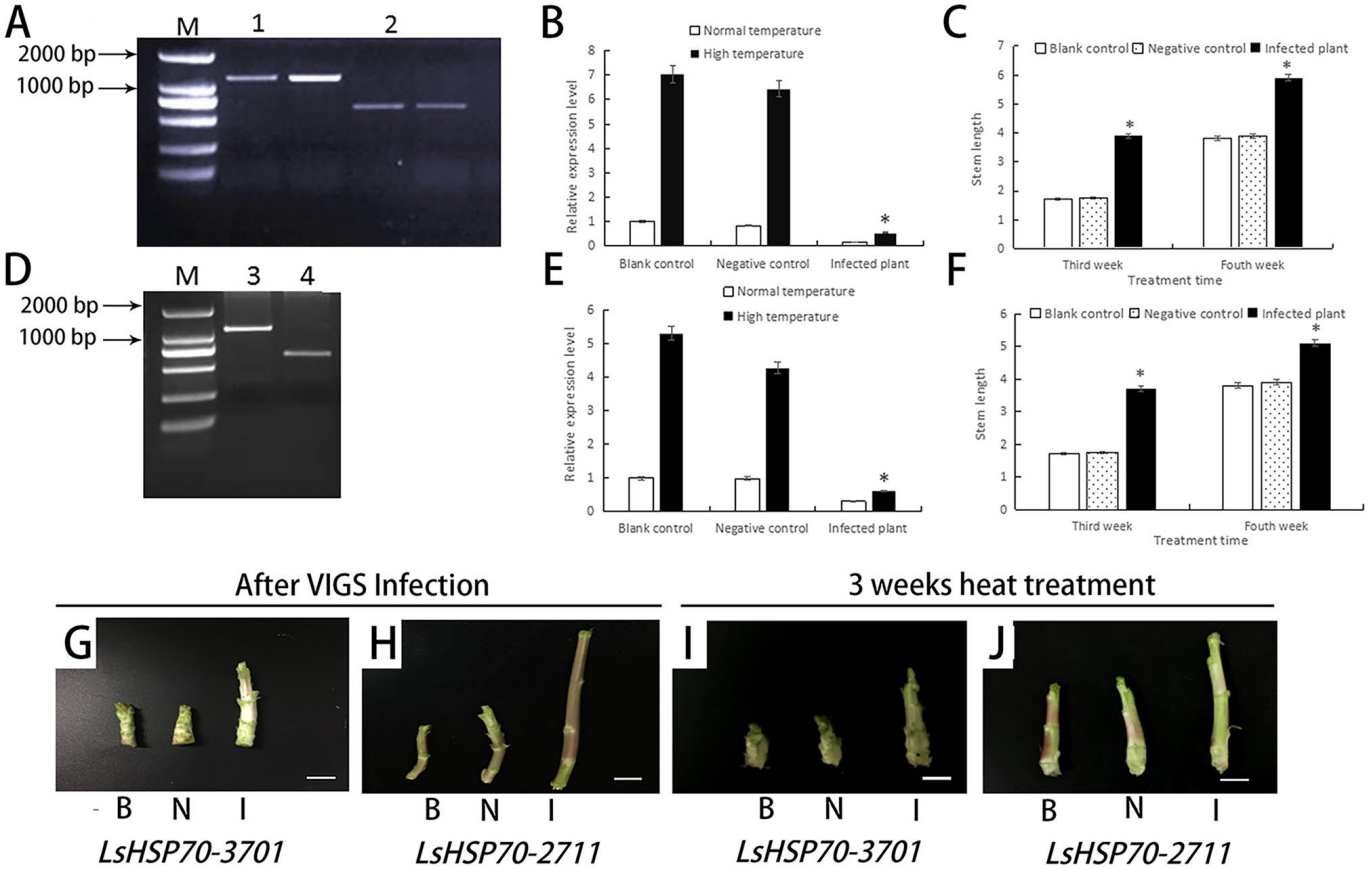
Expression and stem length analysis after infections in lettuce. (**A**,**D**) A TRV detection agarose gel electrophoresis of *LsHsp70-3701I* (**A**) and *LsHsp70-2711* (**D**). Lanes 1 and 3 contain the *LsHsp70-3701* and *LsHsp70-2711* infected plant results, 2 and 4 show the negative controls. (**B**,**E**) Relative levels after infection with *LsHsp70-3701* (**B**) and *LsHsp70-2711* (**E**) in lettuce at room temperature and high temperature. (**C**,**F**) The stem lengths of *LsHsp70-3701* (**C**) and *LsHsp70-2711* (**F**) at 3 weeks after VIGS infection and the corresponding stems at 4 weeks. (**G**–**J**) Lettuce stems of infected plants on week after VIGS infection (**G**,**H**) and 3 weeks after heat treatment (**I**,**J**). Bar = 1 cm. *B* the blank control, *N* negative control, *I* infected plants. Significant differences were determined by Student’s t-test (∗represents P < 0.05).

The cDNAs from the blank control and infected plants were used as templates, and PCR amplification was performed using primers specific to the PTRV2 vector. A band measuring 1,249 bp was detected in the leaves of the *LsHsp70-3701*-infected plants. A band of 1251 bp was detected in the leaves of the plants infected with *LsHsp70-2711*. In both genes, a 754 bp band was detected in the leaves of negative control-infected plants, and no bands were detected in leaves that were uninfected with TRV (Fig. 2A). These results showed that virus-specific bands could be detected in plant leaves, indicating that TRV had initially invaded the leaf cells and had been synthesized, translated and expressed in the cells.

According to the previous analysis, the expression of *LsHsp70-2711* and *LsHsp70-3701* reached a maximum after the 37 °C heat treatment for 60 min and the relative expression of these two genes was compared. The results showed that the expression levels of *LsHsp70-2711* and *LsHsp70-3701* in infected plants both decreased at mRNA level after 3 weeks’ infection (Fig. 2B,C). The relative expression level of *LsHsp70-2711* in infected plants decreased by 68%, comparing with that in the negative control. After high-temperature treatment, the expression in the infected plants showed a decrease of 85% compared with the level in the negative control plants. The relative expression level of *LsHsp70-3701* in infected plants decreased by 82% compared with that in the negative control. Compared with the negative control plants, the infected plants showed a decrease of 91% after high temperature treatment; after the heat treatment, the expression levels of the two genes were both higher than the expression levels of control plants.

There was no significant difference between the negative control and the blank control after 3 weeks of infection. The stem length of the infected plants was significantly different from the stem lengths of the negative and blank controls. Three weeks after infection, the plants were heat shocked for 1 h, and the stem lengths were measured after growing at a normal temperature for 1 week. The results showed that the stem lengths of the infected plants were still significantly different from those of the control plants (Fig. 2D–J).

In conclusion, *LsHsp70-3701* and *LsHsp70-2711* could repress the bolting of lettuce, no matter under control treatment or under heat stress.

### LsHsp70-3701 and LsHsp70-2711 could interact with calmodulin

**Figure 3:**
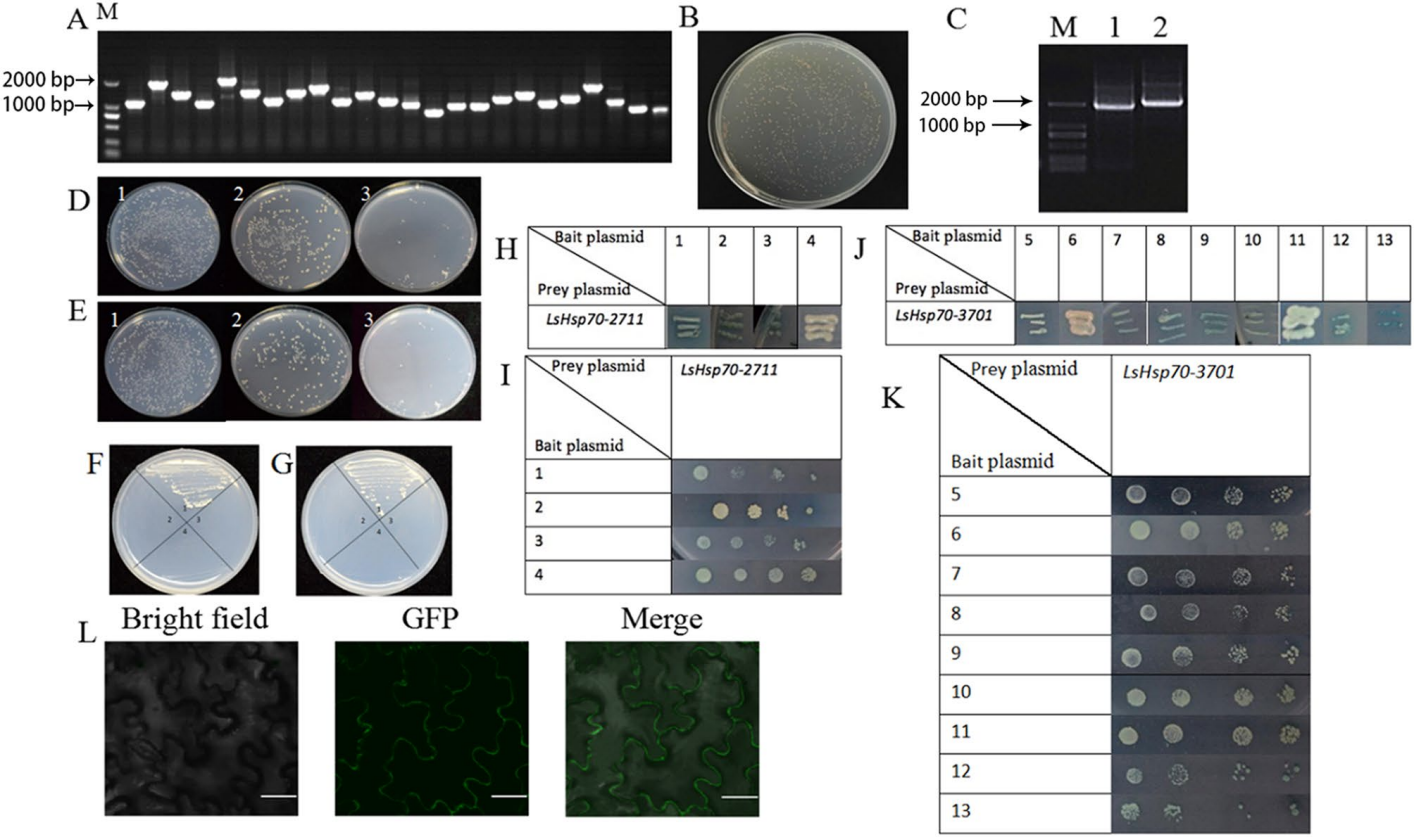
Yeast two-hybrid system. (**A**) The recombination rate of the secondary yeast library inserts was verified. (**B**) A capacity map of the secondary yeast library plate identification library. (**C**) Colony PCR identification by agarose gel electrophoresis. M is the DL 2000 marker, 1 is *LsHsp70-2711* (1944 bp), and 2 is *LsHsp70-3701* (1950 bp). (**D**,**E**) Screening of antibiotic AbA concentrations for PGBKT7-3701 and PGBKT7-2711. The AbA concentrations of 1, 2 and 3 were 75 ng/μl, 87.5 ng/μl and 100 ng/μl, respectively. (**F**,**G**) The bait plasmid self-activation assay. Quadrants 1, 3, and 4 represent the positive controls, negative controls, and PGBKT7; 2 in panel (**F**) represents PGBKT7-3701, and 2 in panel (**G**) represents PGBKT7-2711. (**H**) Yeast two-hybrid screening for prey proteins of *LsHsp70-2711*. (**I**) One-to-one verification of *LsHsp70-2711*. (**J**) Yeast two-hybrid screening for prey proteins of *LsHsp70-3701.* (**K**) One-to-one verification of *LsHsp70-3701*. (**L**) The cell membrane appeared as green fluorescence after the cells were cultured for 48 h. Scale bars represent 50 μm.

The target genes *LsHsp70-3701* and *LsHsp70-2711* were cloned (Fig. 3C). A total of 24 monoclonal clones were identified in the yeast library. The recombination rate was 100% (Fig. 3A). After a 100-fold dilution, the plate was titrated with a titer of 8 × 10^6^ clones (Fig. 3B). Before the yeast screening, different concentrations of AbA were screened to reduce false positives, and the appropriate AbA concentration was 112.5 ng/μl (Fig. 3D,E). A self-activation verification was performed to confirm that the bait plasmid did not self-activate (Fig. 3F,G), and a yeast library screen was performed. The constructed yeast cDNA library was screened for proteins that interacted with the bait plasmid. After primary screening, blue positive monoclonal clones were selected for a secondary screening (Fig. 3H,J) to reduce the false positives. The positive monoclonal clones were selected and expanded to obtain the genes that expressed the interacting proteins (Supplementary Table S1). A one-on-one validation was performed (Fig. 3I,K). Finally, a total of 13 positive library plasmids resulted. Four library plasmid-encoded proteins were confirmed to interact with *LsHsp70-2711* in the yeast two-hybrid system, namely heat shock protein 70, gamma carbonic anhydrase, cyanate hydratase and aspartyl protease. 7 library plasmid-encoded proteins could interact with *LsHsp70-3701* in the yeast two-hybrid system, namely, the calmodulin-binding protein, AP2/ERF domain-containing protein, heat shock protein 70, eukaryotic porin, subtilisin-like protease, C2 calcium-dependent membrane protein, and enolase 2. The calmodulin-binding protein was selected for in vivo validation to demonstrate that Hsp70 does interact with calmodulin-binding protein in plants (Fig. 3L).

## Discussion

### The *Hsp70* genes are of great importance during heat-tolerant bolting in lettuce

An expression analysis of the *LsHsp70* family of 9 lettuce genes showed that the *LsHsp70* genes were induced by high temperature, like other *Hsp70* family genes. For example, in *Arabidopsis*, transgenic experiments showed that cytoplasmic *HSC70-1* can increase the heat tolerance of transgenic plants^25^. Hsp70 is thought to be associated with heat tolerance in rice^26^, and the expression of *HSP* was up-regulated to some extent in rice at high temperatures^27^. Studies have shown that there is a correlation between the expression of *Hsp70* and H_2_O_2_ in maize leaves that is induced by high temperature stress, and H_2_O_2_ promotes the synthesis of Hsp70^28^. On the other hand, Pb stress induced the expression of *Hsp70* and the activity of antioxidant enzymes in broad bean leaves, and Hsp70 was significantly associated with O_2_^29^. VIGS validation showed that the 2 genes are related to heat tolerance and can play an important role in the heat-resistant lettuce during bolting, providing a basis for future experiments.

### A calmodulin that interacts with LsHsp70 was obtained from lettuce

In this study, we performed four repeated screenings of *LsHsp70-2711* and *LsHsp70-3701*. The genes were verified by strict screening and one-to-one hybridization. Based on the self-activation of library plasmids, 13 positive library plasmids were ultimately screened. 4 library plasmid-encoded proteins could interact with *LsHsp70-2711* in a yeast two-hybrid system, and 7 library plasmid-encoded proteins could interact with *LsHsp70-3701* in a yeast two-hybrid system, including calmodulin, calcium-dependent proteins and homologous proteins (Fig. 3H–K, Supplementary Table S2). Studies have shown that Ca^2+^ can affect the heat tolerance of maize germ and the levels of hormones. Furthermore, Ca^2+^ and calmodulin are involved in the heat stress response of maize^30^. In Klein’s study^31^, when cells in pears were suspended, the concentration of calcium in the cytoplasm could be increased by high-temperature stimulation on a linear correlation trend. Meanwhile, the increase in Hsp-72 and its mRNA content in human A-431 epidermal cells primarily depended on the increase in the Ca^2+^ concentration and the increase in the G protein^32^. Heat treatment of transgenic tobacco (MAQ 2.4) led the Ca^2+^ concentration in the cytoplasm increase at first and then decrease, confirming that the heat shock response in tobacco was related to the calcium ion concentration^33^. Therefore, we hypothesize that the heat resistance mechanism of LsHsp70 is related to the calcium-calmodulin pathway and is directly related to the Ca^2+^ concentration and calmodulin. In our study, it is already known that HSP70 has a molecular chaperone function. Following yeast two-hybrid system verification, we found that Hsp70 interacted with other proteins and activated downstream gene expression, thereby improving the heat resistance of lettuce. In this case, it would be possible to continue the exploration in the heat resistance of lettuce. It is of great significance to study how Hsp70 binds to the calcium-calmodulin pathway in order to improve the heat tolerance of plants. However, more studies focusing on the interaction between Hsp70 and calmodulin, like Co-IP and GST-pull down, should be carried out to verify the results in the future.

### High temperature promotes the interaction between calmodulin and Hsp70, inducing GA accumulation and resulting in bolting

According to previous researches, GA could significantly promote the bolting of lettuce, and played leading role in the bolting of radishes^20,21^. In *Arabidopsis*, the cGMP and GA response element (GARE) plays an important role in GA signal transduction^22^. Therefore, it can be hypothesized that the high temperature could enhance the accumulation of GA in the lettuce.

On the other hand, high temperature induced an increase in the gene expression of *LsHsp70*. Yeast two-hybrid method was applied to obtain a calmodulin protein that can interacted with *LsHsp70-3701* and high temperature which could promote the accumulation of GA, leading to bolting. In *Arabidopsis*, the regulation of GA plays an important role in bolting^4^. Tobacco plants expressed a large amount of *NtCBK2* in response to GA stress, indicating that *NtCBK2* expression may be related to GA regulation^34^. Also, Ca^2+^ influx and the action of Ca-dependent protein kinases were known to be closely interrelated with the expression of HSPs^35^. As a mediator of Ca^2+^ signalling, CaM can be activated by binding Ca^2+^, inducing the regulation of many HSP genes^36^. On the other hand, Ca^2+^ has been shown to be involved in the regulation of plant responses to high temperature^18^.

Therefore, we hypothesize that after a high-temperature exposure, lettuce produces GA and its heat shock proteins start to interact with calmodulin in order to resist heat stress. The lettuce hormone regulation pathway may then be activated. However, GA_3_ accumulates again and reaches its peak later, which could promote the bolting in lettuce as a result.

Thus, heat shock proteins have a great influence on the calmodulin pathway and hormone regulation through direct protein interaction, but they may also have indirect effects on the calpain pathway and the hormone pathway. We can speculate on three additional matters specifically, for some inquiries: whether these pathways work together, how the proteins perform their functions and whether more protein interactions are important, and whether other small molecules such as RNA and DNA, are involved. All these inquiries require us to use other technologies, such as the yeast three-hybrid approach. However, these questions will allow us to explore the next steps in understanding the functions of heat shock proteins.

## Conclusions

In this study, we obtained *LsHsp70-3701* and *LsHsp70-2711* under high temperature treatment of lettuce. The VIGS system was used to verify that the two genes were related to the heat-tolerant bolting of lettuce. By applying yeast two-hybrid technology, we found that Hsp70 can interact with calmodulin at high temperature. Also, it was shown that GA can accumulate under high temperature, while the GA content had an effect in promoting bolting. This study lays the foundation for the comprehensive heat-resistance study of lettuce.

## Supplementary information

Supplementary Information
