# Supplementary material for: LsHSP70 is induced by high temperature to interact with calmodulin, leading to higher bolting resistance in lettuce
Source: Sci Rep. 2020 Sep 16;10:15155. doi: 10.1038/s41598-020-72443-3 (PMC7495476; doi:10.1038/s41598-020-72443-3)
Supplement: Supplementary file 1 — Supplementary Information [file 41598_2020_72443_MOESM1_ESM.pdf]

LsHSP70 is induced by high temperature to interact with calmodulin, leading to higher bolting resistance in lettuce

Ran Liu [1+], Zhenqi Su[1+], Huiyan Zhou[2+], Qian Huang[1], Shuangxi Fan[1], Chaojie Liu[1], Yingyan Han[1*]

1. *Beijing Key Laboratory of New Technology in Agricultural Application, National Demonstration Center for Experimental Plant Production Education, Beijing University of Agriculture, Beijing, 102206, China*
2. *Laboratory of Cell & Molecular Biology, Institute of Vegetable Science, Zhejiang University, Hangzhou, 310058, China*

+Co-first authors.

*Correspondence: hyybac@126.com (Han Y.) Tel: 8610-81798039.

Received: date; Accepted: date; Published: date

Legends for supplementary materials:

Supplementary Table S1 Sequences of primers used in this work

Supplementary Table S2 Interacting protein genes

Supplementary Table S1 Sequences of primers used in this work

Primer name	Primer sequence（5'–3'）
PGBKT7-3701F	CCCATATGGGGGGTGATCGGAAATATGTCG
PGBKT7-3701R	GGCCNNNNNGGCCCTCGATCACCCTTAATCCAC
PGBKT7-2711F	CCCCGGGGATGGCCGGTAAAGGCGAAGGTCCCG
PGBKT7-2711R	TTGCGGCCGCAATTACTGTCGACCTCCTCGATCTTAGGGCCA
VIGS-3701F	GTGAGTAAGGTTACCGAATTCTCTAGAAACACCACAATTCCGACCAAG
VIGS-3701R	GGGCCTCGAGACGCGTGAGCTCGGTACCCCGCATCCTCCACCTTCTT
VIGS-2711F	CCTGGAGTGTTGATCCAGGTGTACG
VIGS-2711R	CTCAAACCACAATCGAGATTGACTC
VIGS- F	CGCACGTATGAAGTAATTGGA
VIGS- R	GCCAAATGTTTGAACGATCGG
3701Y-F	CTAAAGACAACAACCTGCTCGG
3701Y-R	CTGCACCATCTTCTCTATCTCCTC
2711Y-F	GACTTGTTGCTATTGGATGTCA
2711Y-R	TATCCCTCGTTCTTGTCCTTTC
Lshsp70-2711-F	AGGATGCGATTGATGAGGCGATTG
Lshsp70-2711-R	CACCTGCTCCACCTTGATACATCTTC
Lshsp70-3701-F	TCCTGCTCCTCGTGGTGTTCC
Lshsp70-3701-R	CTCTATCTCCTCCTTCGACAGTCTCC
18SR-F	GTGAGTGAAGAAGGGCAATG
18SR-R	CACTTTCAACCCGATTCACC
3701-YCE-F	GCCACTAGTGGATCCATGTCGGGGAAAGGTGAAG
3701-YCE-R	AGCGGTACCCTCGAGATCCACCTCTTCAATCTTGGGTC
YNE-Ca-F	CCTACTAGTGGATCCATGGGTAAGACAACCAGGTGGC
YNE-Ca-R	AGCGGTACCCTCGAGCCTCCGAAAAGCCCTCTCAG

Supplementary Table S2 Interacting protein genes

number	Gene	Protein
1	Last_1_v5_gn_9_22920.1	PLY93073.1
2	Last_1_v5_gn_5_27781.1	PLY73555.1
3	Last_1_v5_gn_1_75161.1	PLY70610.1
4	Last_1_v5_gn_7_31721.1	PLY74619.1
5	Last_1_v5_gn_2_131600.1	PLY68769.1
6	Lsat_1_v5_gn_4_52000.1	PLY63948.1
7	Last_1_v5_gn_8_78021.1	AKN91187.1
8	Last_1_v5_gn_2_83381.1	PLY94517.1
9	Last_1_v5_gn_5_122520.1	PLY80633.1
10	Last_1_v5_gn_2_130220.1	PLY92266.1
11	Last_1_v5_gn_5_53581.1	PLY71974.1
12	Last_1_v5_gn_4_158640.1	PLY68010.1
13	Last_1_v5_gn_2_21500.1	PLY61647.1
